# Results of cytology and high-risk human papillomavirus testing in females with cervical adenocarcinoma *in situ*

**DOI:** 10.3892/ol.2013.1350

**Published:** 2013-05-15

**Authors:** SONIA ANDERSSON, MIRIAM MINTS, ERIK WILANDER

**Affiliations:** 1 Department of Women’s and Children’s Health, Division of Obstetrics and Gynecology, Karolinska University Hospital-Solna, The Karolinska Institute, Stockholm S-171 76;; 2 Departments of Genetics and Pathology and Clinical Pathology and Cytology, Rudbeck Laboratory, Uppsala University Hospital and Uppsala University, Uppsala S-751 85, Sweden

**Keywords:** cervix, adenocarcinoma *in situ*, HPV, screening, Pap smear, CIN

## Abstract

The incidence rates of cervical adenocarcinoma have been increasing over the last two decades, contrary to those of squamous cell carcinoma. This trend is particularly evident among females aged <40 years and has occurred despite extensive cytology-based screening programs. The aim of the present retrospective database study was to investigate adenocarcinoma *in situ* (AIS) with respect to previous cytological results, high-risk (HR) human papillomavirus (HPV) infections and histological results from AIS-adjacent squamous mucosa. Databases were used to identify 32 female patients with AIS treated for various conditions between 2009 and 2012 at the Department of Gynecology, Uppsala University Hospital (Uppsala, Sweden) and previous cytological, HPV and histological results. Of the individuals in the study, 64.3% had a previously recorded cytological result showing squamous cell abnormalities; five had glandular cell abnormalities (18%) and two had AIS (7.1%). Among the patients with available HPV results, 95% were HR-HPV-positive; HPV18/45 predominated (77%), followed by HPV16 (27%). The patients with multiple HPV infections were aged ≤32 years, while patients aged ≥38 years were only infected with HPV18/45. All but three patients had cervical intraepithelial neoplasia (CIN) in the AIS-adjacent squamous mucosa, 79% of which was CIN2 or worse. The present retrospective database study suggests that AIS is detected at screening mainly due to simultaneous squamous precursor lesions and that HPV18/45 infection is an increasing cofactor for AIS in older patients. HPV analyses of glandular precursor lesions aid in the identification of female individuals at risk of progression to invasive disease, and thus have a favorable effect on adenocarcinoma prevention, together with vaccination.

## Introduction

Cervical cancer is a disease that mainly affects females in the fourth or fifth decade of life. According to the International Agency for Research on Cancer, cervical cancer is the second most common cancer in females worldwide and the most common in low- and middle-income countries (1). High-risk (HR) human papillomavirus (HPV) is a significant etiological agent in the pathogenesis of cervical squamous cell carcinoma (SCC) and adenocarcinoma (2–4). HPV DNA is detected in 90% of adenocarcinomas, with HPV18 being the predominant type (5,6). Incidence rates of adenocarcinoma, which accounts for 10–20% of all cervical cancers, have been increasing in high-income countries for the last two decades, unlike those of SCC (7,8). This upward trend is particularly evident among females <40 years of age and has occurred despite the existence of extensive cytology-based cervical cancer screening programs (9,10).

Although screening programs have led to a substantial decrease in the incidence of SCC, they have had only a limited protective effect on the occurrence of adenocarcinoma and its precursor lesion, adenocarcinoma *in situ* (AIS) (11). AIS is a relatively rare condition that has not gained as much attention as precursor lesions with an origin in the squamous epithelium of the cervix. AIS is known to occur beneath the transformation zone and may thus be covered by normal metaplastic or dysplastic epithelium. Furthermore, the multicentricity of AIS has been reported in ∼15% of patients (12). However, a nationwide study conducted in Sweden indicated that cytology screening is of value for detecting adenocarcinoma at an early stage (13). HPV testing is more sensitive than cytology as a screening method for SCC (14–16) and the early detection of HPV DNA has also been suggested as an alternative screening method for adenocarcinoma and AIS (17,18).

The aim of the present study was to investigate AIS with respect to cytological results, HPV infections and histological results from the AIS-adjacent squamous mucosa using data from the SymPathy database of the Department of Pathology, Uppsala University Hospital (Uppsala, Sweden) and the database of the organized cervical cancer screening program of the County of Uppsala.

## Materials and methods

The SymPathy database (Tieto AB, Malmö, Sweden) contains information on results obtained at the Department of Clinical Pathology and Cytology, Uppsala University Hospital. Data therein are identified by specific topographical and morphological codes [Systematized Nomenclature of Medicine (SNOMED); College of American Pathologists, Skokie, IL, USA]. The database of the organized population-based cervical cancer screening program of the County of Uppsala includes cytological, HPV and histological results from all laboratories in the county. Cytological and histological samples from the screening program are stored at the Department of Clinical Pathology and Cytology, where all cytological, HPV and histological testing for the screening program is performed. The study was approved by the Regional Ethical Review Board in Uppsala, Sweden.

A total of 32 female patients with histologically confirmed AIS, who received treatment for various conditions at the Department of Gynecology at Uppsala University Hospital between 2009 and 2012, were identified in the SymPathy database, and the previous cytological, HPV and histological results for these patients were retrieved from the database of the cervical cancer screening program of the County of Uppsala. Cytological results were available for 28 patients, two of whom had provided cytological samples due to clinical symptoms (vaginal bleeding). HPV results were available for 22 patients, of whom three had participated in primary screening with HPV tests (19) and one obtained results from a self-sampling of vaginal fluid at home (20). Histological results were available for all 32 patients, including for one in whom AIS was identified following a hysterectomy due to an ovarian tumor.

All the cytological results in the database were classified according to the classification of cervical intraepithelial neoplasia (CIN) by the Swedish Society for Clinical Cytology (21). For the purposes of the present study, the cytological results were reclassified using the Bethesda system, excluding koilocytosis without nuclear atypia from the results of low-grade squamous intraepithelial lesions (LSIL) (22).

The HPV results in the database were obtained by applying cervical cells to an indicating FTA elute card (filter paper matrix, art. no. WB120411; Whatman, Inc., Clifton, NJ, USA) (23,24) for HPV detection performed using an hpVIR multiplex real-time PCR assay. Detailed descriptions of the detection of the HPV status and HPV screening analysis results have been published previously (24). Briefly, a 3-mm diameter Harris micro-punch (GE Healthcare, Little Chalfont, UK) was used to excise four sections from the FTA cartridge and the FTA cards using a BSD 600 (BSD Robotics, Queensland, Australia) semi-automatic punch robot. The four punches from each sample were transferred to a single well in a 96-well plate and washed by vortexing three times for 5 sec in 200 *μ*l distilled H_2_O. The water was then carefully removed with a pipette. The DNA elution was performed in 50 *μ*l distilled H_2_O at 95°C for 30 min in a heating block (with heated lid). The DNA extract (3 *μ*l) was used as the template in each real-time PCR (24).

HPV typing was performed as described previously, using a real-time PCR-based assay (23,25). This assay detects and quantifies a human single copy gene [house keeping gene; *Homo sapiens* hydroxymethylbilane synthase (HMBS); GenBank accession no. M95623.1] and the following HPV types, partly in groups: 16, 18/45, 31, 33/52/58, 35, 39, 51, 56 and 59. In order to be able to determine if a sample contained sufficient amounts of material for an HPV test to be considered informative, a threshold of 10 copies of the nuclear single copy gene was used per PCR, based on the analysis of HMBS. In addition, for the HPV test itself, the sample had to contain a minimum of 10 HPV copies to be typed positive (24).

All 32 patients had histological results available in the database from either punch biopsies, conization or hysterectomies. The histological samples of the 32 patients were re-evaluated by the local pathologist and classified according to the ICD-10 (26). AIS was diagnosed in the cone resections of 27 patients, in the hysterectomy specimens of three patients and in the cervical punch biopsies of two patients. One patient who underwent conization due to a diagnosis of AIS from population-based cervical cancer screening, was diagnosed with invasive adenocarcinoma in the cone resection (case 32). The most severe grade of atypia observed in each biopsy determined the histological diagnosis.

## Results

In total, 18/28 (64.3%) patients with AIS and an available cytological result showed signs of squamous cell changes, including 10 patients with atypical squamous cells of undetermined significance (ASCUS), three with LSIL and five with high-grade squamous intraepithelial lesions (HSIL). Only five patients had a cytological result of atypical glandular cells of undetermined significance (AGUS, 17.9%) and two exhibited AIS (7.1%; Table I).

The HPV tests recorded in the database were performed on average 4.5 months (range, 0–12 months) prior to the histological AIS diagnosis. A total of 22 patients were tested for HPV, 95% of whom were HR-HPV-positive. HPV18/45 was markedly predominant and occurred in 77% (n=17) of the HPV-positive patients, followed by HPV16 (27%, n=6). Multiple HPV infections were recorded in five patients; three had double infections (HPV16+18/45, HPV18/45+59 and HPV18/45+59) and two exhibited triple infections (HPV16+18/45+33/52/58 and HPV16+33/52/58+56). The patients with multiple infections were all ≤32 years of age, while the patients aged ≥38 years were only infected with HPV18/45.

The AIS-adjacent squamous mucosa showed various grades of CIN in all but three of the 32 patients with AIS, in whom the squamous epithelium did not show signs of atypical cells. Notably, CIN2 or worse was identified in the AIS-adjacent squamous mucosa of 25 patients with AIS (78.1%; Table I).

## Discussion

Cervical carcinogenesis is a stepwise process. Between 15 and 20 years are usually required for the increasingly abnormal (dysplastic) cervical epithelial lesions to progress to invasive cancer. Persistent infection has a critical role in the oncogenic HPV types (27–30). Since the glandular epithelium of the cervix is a monolayer, there is no such stepwise division of potentially precancerous lesions. The precancerous lesions that arise from these cells are called AIS or glandular intraepithelial neoplasia (GIN). In the present study, an extremely high percentage (95%) of HR-HPV-positivity was observed among the patients with AIS. HPV types 18/45 were clearly predominant and occurred in 77% of study patients, followed by HPV16 (27%). There have been few investigations of the prevalence of HPV in GIN, although the present study results suggest that HPV DNA may be detectable in 70–85% of AIS, indicating that adenocarcinoma and SCC have a close pathogenic association.

A previous study identified a special target cell in the junction between the glandular and squamous cells, which is highly susceptible to oncogenic HPV DNA incorporation (31). It is known that SCC mostly contains HPV16 and adenocarcinoma mostly HPV18 (6). Therefore, it is possible that a type of tropism exists with regard to the oncogenic potential of the various HPV types and that in addition to initiating malignant transformation, the oncogenic HPV types also affect the final malignant cell differentiation of the target cells. AIS is recognized as a precursor lesion of invasive adenocarcinoma due to its morphological futures, which are similar to those of invasive adenocarcinomas. A study reported that the same HPV types were detected in AIS and in the AIS-adjacent squamous mucosa, demonstrating that AIS is a precursor lesion that may progress to invasive adenocarcinoma (32). The present study also supports this hypothesis. In a study by Dahlström *et al* (18), it was shown that HPV type-specific risks for AIS and adenocarcinoma are not the same as for SCC, with HPV18 being important and other oncogenic types having little or no association. These findings are in accordance with the present study, in which HPV18 was consistently observed to be preferentially associated with AIS. However, while Castellsagué *et al* reported an association between cervical adenocarcinoma and HR-HPV types other than HPV16 and 18 (4), Dahlström *et al* concluded that other oncogenic HPV types were not associated with risks for AIS or adenocarcinoma in a Swedish cohort (18). Furthermore, AIS is contiguous to invasive adenocarcinoma and often AIS incidence peaks 5–8 years prior to the diagnosis of invasive adenocarcinoma (32). In the present study, the frequent occurrence of CIN, detected in 79% of AIS-adjacent squamous tissue, was a notable observation. The pathologist often observed AIS coexisting with squamous cell abnormalities. Careful surveillance is required for females with multiple lesions due to the fact that invasive adenocarcinoma takes fewer years to develop than SCC and therefore these individuals are at a higher risk of developing invasive carcinoma than those with preinvasive squamous lesions of the cervix only.

Cytological screening has reduced the incidence of SCC (33), whereas the incidence of adenocarcinomas is increasing (33,34). This may indicate that cytological screening is unable to identify females at risk of adenocarcinoma. However, it has been shown that cytological screening is effective against adenocarcinomas (13). In agreement with the latter observation, adenocarcinomas have been shown to be displayed in a high percentage of co-existing CIN lesions, which may be identified by cytological screening. In the present study, AIS was detected in 64.3% of the patients due to accompanying squamous abnormalities, but in only 27% due to glandular abnormalities. This may be due to the relatively low sensitivity of cytology screening (35), which has been ineffective in reducing the incidence of adenocarcinomas. The reason for this low sensitivity may be the difficulties in obtaining cells in the endocervical canal or non-specific misreading of glandular lesions. Liquid-based cytology may provide more accurate diagnoses of glandular disease since the preparation allows a monolayer of cells to form and interpretation errors are reduced (36).

In a previous study in Stockholm (Sweden) the percentage of adenocarcinomas of the uterine cervix varied between 2 and 7% across a prescreening cohort. During the screening period, adenocarcinoma cases increased significantly and accounted for 25.7% of cases on average (P<0.001), with a marked difference observed between younger and older individuals. In the screening cohort, the percentages of adenocarcinoma among younger patients and patients ≥70 years were approximately six- and three-fold higher, respectively, compared with the prescreening cohort (37).

Although the present study was based on a relatively small number of cases, it is notable that the relative proportion of HPV18/45 infections in AIS increased with age. All patients ≥38 years of age were only infected with HPV18/45. In addition, the majority of patients ≥44 years of age were admitted for colposcopy due to a positive HPV test or a cytological result of abnormal glandular cells, not atypical squamous cells. The reason for this age-related difference remains unclear.

It is noteworthy that almost all patients with AIS had a recorded HPV infection with an oncogenic type. Cytology screening has a low sensitivity for identifying pre-malignant squamous alterations compared with primary HPV testing. It has been reported that HPV testing may be as much as twice as sensitive as cytology and that in older patients the difference may be even more pronounced (35,38). When taking this into consideration, it is tempting to speculate that primary HPV testing for the prevention of cervical squamous carcinomas has a corresponding significance for the prevention of adenocarcinomas, as has previously been indicated (4,39,40). Dahlström *et al* showed that infections with HPV16 and 18 detected 15 years prior to diagnosis when no cytological changes were evident, were associated with an increased risk of developing *in situ* and invasive cervical adenocarcinoma (18). Detection of HPV16 and 18, as objective molecular markers, may allow a diagnosis of cervical adenocarcinomas at an earlier stage in the natural history of the disease. Furthermore, HPV analyses of the precursor lesions, AIS and AGUS, may aid in the identification of patients at risk of further progression to invasive disease, thus having a favorable effect on the prevention of adenocarcinomas together with vaccination against HPV16 and 18.

## Figures and Tables

**Table I.** Results of cytology screening, HR HPV testing and histology of adjacent squamous mucosa in association with cervical AIS.

Cases	Age (years)	Cytological results	Squamous mucosa	HPV types
1	23	HSIL	CIN 3	16+18/45
2	23	LSIL	CIN 3	-
3	25	LSIL	CIN 1	16
4	26	LSIL	CIN 3	16+18/45+33/53/58
5	26	ASCUS	CIN 2	18/45+59
6	26	ASCUS	CIN 1	16+33/53/58+56
7	30	ASCUS	CIN 1	-
8	31	ASCUS	CIN 3	18/45
9	32	ASCUS	CIN 3	18/45+59
10	32	WNL	CIN 3	-
11	32	WNL	CIN 2	-
12	32	AGUS	CIN 3	-
13	32	ASCUS	CIN 1	18/45
14	33	AGUS	CIN 3	18/45
15	33	AGUS	Normal	18/45
16	34	HSIL	CIN 3	16
17	36	ASCUS	CIN 2	16
18	38	ASCUS	CIN 3	18/45
19	39	HSIL	CIN 1	-
20	40	AGUS	CIN 1	18/45
21	41	ASCUS	CIN 3	Negative
22	42	ASCUS	CIN 3	18/45
23	42	AIS	CIN 3	18/45
24	43	AGUS	Normal	18/45
25	44	Not available	Normal	-
26	44	HSIL	CIN 3	18/45
27	44	HSIL	CIN 2	-
28	44	AGUS	CIN 3	18/45
29	46	Not available	CIN 3	-
30	47	AIS	CIN 1	-
31	60	Not available	CIN 3	18/45
32	61	Not available	CIN 3	18/45

HPV, human papillomavirus; AGUS, atypical glandular cells of undetermined significance; AIS, adenocarcinoma *in situ*; ASCUS, atypical glandular cells of undetermined significance; LSIL, low-grade squamous intraepithelial lesions; HSIL, high-grade squamous intraepithelial lesions; CIN, cervical intraepithelial neoplasia; WNL, within normal limits.
